# Current application and future perspectives of antimicrobial degradable bone substitutes for chronic osteomyelitis

**DOI:** 10.3389/fbioe.2024.1375266

**Published:** 2024-03-27

**Authors:** Chenxi Jiang, Guangxun Zhu, Qian Liu

**Affiliations:** ^1^ Department of Stomatology, Union Hospital, Tongji Medical College, Huazhong University of Science and Technology, Wuhan, China; ^2^ School of Stomatology, Tongji Medical College, Huazhong University of Science and Technology, Wuhan, China; ^3^ Hubei Province Key Laboratory of Oral and Maxillofacial Development and Regeneration, Wuhan, China; ^4^ Department of Stomatology, Tongji Hospital, Tongji Medical College, Huazhong University of Science and Technology, Wuhan, China

**Keywords:** chronic osteomyelitis, regenerative bone substitutes, infection eradication, bone reconstruction, antimicrobial degradable bone substitutes

## Abstract

Chronic osteomyelitis remains a persistent challenge for the surgeons due to its refractory nature. Generally, treatment involves extensive debridement of necrotic bone, filling of dead space, adequate antimicrobial therapy, bone reconstruction, and rehabilitation. However, the optimal choice of bone substitute to manage the bone defect remains debatable. This paper reviewed the clinical evidence for antimicrobial biodegradable bone substitutes in the treatment of osteomyelitis in recent years. Indeed, this combination was proved to eradicate infection and facilitate bone reconstruction, which might reduce the cost and hospital stay. Handling was associated with increased risk of unwanted side effect to affect bone healing. The study provides some valuable insights into the clinical evaluation of treatment outcomes in the aspects of infection eradication, bone reconstruction, and complications caused by materials. However, achieving complete infection eradication and subsequently perfect bone reconstruction remains challenging in compromised conditions, hence advanced innovative bone substitutes are imperative. In this review, we mainly focus on the desired functional effects of advanced bone substitutes on infection eradication and bone reconstruction from the future perspective. Handling property was optimized to simplify surgery process. It is expected that this review will provide an important opportunity to enhance the understanding of the design and application of innovative biomaterials to synergistically eradicate infection and restore integrity and function of bone.

## 1 Introduction

Osteomyelitis can be defined as bone inflammation caused by an infectious agent. There are three clinical mechanisms that result in bone infection: acute hematogenous osteomyelitis, which is more common in pediatric patients; osteomyelitis secondary to vascular insufficiency or neuropathy; and osteomyelitis resulting from the spread of a contiguous source trauma or surgical contamination (Urish and Cassat, 2020). In adult patients, it is estimated that 47%–50% of all osteomyelitis cases are post-traumatic (Lew and Waldvogel, 2004; Lima et al., 2014). Chronic osteomyelitis represents a major health problem as its significant morbidity and refractory nature, despite advances in current healthcare (Hatzenbuehler and Pulling, 2011). Additionally, chronic osteomyelitis is usually sustained for more than a month. In a worst case scenario, osteomyelitis can become a lifelong disease with late reactivation up to 80 years after the primary episode (Kremers et al., 2015).

One of the main obstacles for osteomyelitis treatment is that the infectious agent can remain in the bone tissue for a long period of time (Lew and Waldvogel, 2004). Since the inflammation actually takes place in avascular zones, the bactericidal efficacy of systemic antibiotics administration and the host immune system are spatially diminished. Given this, local release of antibacterial agents represents an appealing choice for the treatment of chronic osteomyelitis. Historically, antibiotic-loaded acrylic cement (ALAC) (Alonso et al., 2019), polymethylmethacrylate (PMMA) beads (Wentao et al., 2017), and calcium sulfate pellets (Chang et al., 2007; McKee et al., 2010; Gauland, 2011; McNally et al., 2016; Badie and Arafa, 2019; Qin et al., 2019) have outpaced systemic antibiotic therapy in Europe. However, ALAC and PMMA are nonbiodegradable. Patients suffer from secondary surgery for removal of the polymeric devices after antibiotic release, increasing the cost and duration of hospitalization. Without surgical removal, such devices may generate foreign bodies, thereby inducing subsequent inflammation and infection (Gibon et al., 2017). Consequently, an optimal device should provide the sustained release of local antimicrobial agent. Secondly, the device should not jeopardize the process of new bone ingrowth and preferably promote bone regeneration. As such, resorbable osteoconductive biomaterials integrated with antimicrobial agents seem appealing for one-stage surgery in the treatment of osteomyelitis.

The purpose of this review is to provide an overview of recent research on degradable bone substitutes integrated with antimicrobial agents for the treatment of chronic osteomyelitis, including experimental work as well as efforts that have already progressed into a clinical stage. For this, an outlook to a new versatile generation of materials is given to heal the refractory osteomyelitis, with the aims of improving quality of life and reducing hospital readmission.

## 2 What is chronic osteomyelitis?

As the name suggests, chronic osteomyelitis becomes symptomatic for a long duration. It occurs mainly after a fracture and occasionally due to ischemic ulcers caused by diabetes mellitus, sickle cell disease, and malnutrition. The differentiation diagnosis to distinguish from acute infection is dead bone formation and host reparative reactions. Some devitalized bone fractures after trauma and impairment of the vasculature contribute to bone sequestrum. Host reactive new bone formation around the sequestrum occurs in the changeable course, defined as involucrum (Walter et al., 2012). Sequestrum colonized by bacteria aggravates the inflammatory response. The main pathogenic bacterium responsible for osteomyelitis is *Staphylococcus aureus (S. aureus).* Its virulence factors subvert host immune defense and culminate in bone destruction. For instance, *S. aureus* protein A (spA), an extracellular and cell-bound protein, has been documented to induce severe inflammatory response (Kumar et al., 2007), inhibit osteogenesis, and induce osteoclastogenesis (Jin et al., 2013). In addition, more than 50% of cases were related to hard-to-treat Methicillin-resistant *Staphylococcus aureus* (MRSA) (Gallarate et al., 2021).

Chronic osteomyelitis may frequently relapse, which is attributed to the imbalance between the host’s defense and bacterial invasion. Deficiency in local immune response hampers the clearance of the infection agents. Local ischemia prevents the infiltration of inflammatory cells and systemically administered antibiotics into this avascular region. Consequently, a biofilm is formed on the sequestrum, which protects the inner bacteria from antibiotics, host immune defense cells and the penetration of antibodies. The sessile form of pathogens within the biofilm reduce the sensitivity to antibiotics by a factor of 10^3^, compared with a planktonic phase (Walter et al., 2012). On the other hand, *S. aureus* can develop an altered bacterial phenotype with a very slow metabolic rate, referred to as small colony variant (SCV). The bacterium can penetrate the host cells and survive intracellularly as a slow, persistent, and indolent infection, which is largely insensitive to antibiotics (Lehar et al., 2015; Tuchscherr et al., 2015). Meanwhile, the bacteria from the sessile phase in the biofilm could return to the planktonic phase. Clinically, this phenomenon would further trigger the recurrence of osteomyelitis once host defense system is inhibited. Therefore, chronic osteomyelitis remains a difficult clinical condition to treat, requiring multiple surgeries and a prolonged hospital stay.

## 3 What is the current standard of care?

The comprehensive treatment of chronic osteomyelitis requires a multimodal approach involving adequate antimicrobial therapy, surgery, bone reconstruction, and rehabilitation (Barakat et al., 2019). Antimicrobial therapy plays an adjunctive role as the pathogens are tolerate to antibiotics. Antimicrobial therapy can be empirical or definite. Occasionally, empirical antimicrobial therapy is required initially before the antibiogram test based on cultures obtained from bone biopsies. Patient-specific consideration is essential involving drug allergy, drug toxicity, and drug metabolism, etc. Definite antimicrobial therapy is mainly based on microbiological diagnosis and susceptibility testing against the causative pathogen, if possible. Crucial microbiological sampling of the deep infection occurs at the time of initial debridement, avoiding iatrogenic bacterial contamination as carefully as possible. The choice of antibiotic therapy should be tailored to the individual patient based on susceptibility. Other factors such as disease chronicity and progress, patient compliance, and overall health with an evaluation of systemic markers of inflammation should be also considered (Gallarate et al., 2021). Penicillin remains the first-choice antibiotic. Clindamycin, metronidazole, ticarcillin and clavulanic acid, cephalosporins, carbapenems, vancomycin in association with other antibiotics could be chosen for resistant microorganisms. Empirically, a complete course of parenteral antibiotic therapy is prescribed over 4–6 weeks. Alternatively, initial parenteral therapy is followed by 3 weeks of oral antibiotics, such as ciprofloxacin and levofloxacin, which have excellent oral bioavailability and bone penetration (Huang et al., 2019). There are some recommended regimens for osteomyelitis antibiotic therapy targeting different types of microorganisms (Bury et al., 2021; Gallarate et al., 2021). For example, penicillin was recommended to use every 24 h as first-line Intravenous treatment against penicillin-sensitive *S. aureus*, while as for penicillin-resistant *S. aureus,* nafcillin or oxacillin was recommended to use every 4–6 h. Moreover, the Infectious Disease Society of America (IDSA) and the European Society for Pediatric Infectious Disease (ESPID) provide clinical practice guidelines for osteomyelitis treatment (Liu et al., 2011; Serrano et al., 2020). For MRSA induced osteomyelitis, the IDSA recommends empirical therapy with vancomycin or daptomycin plus rifampin for the initial 2 weeks, followed by rifampin plus another oral agent (fluoroquinolone, trimethoprim-sulfamethoxazole, a tetracycline, or clindamycin) to complete 3–6 months of therapy. ESPID guidelines recommends clindamycin with or without an anti-*staphylococcal* beta-lactam. For children with severe infection, vancomycin is a preferred choice, with or without the inclusion of clindamycin or an anti-*staphylococca* beta-lactam (Urish and Cassat, 2020).

Antibiotics should be able to reliably penetrate bone and combat against the expected pathogen spectrum effectively. The penetration ability of antibiotic into bone is evaluated by calculating the ratio of bone-to-serum concentration (mg/kg to mg/L). Azithromycin (ratio of bone-to-serum concentration: 2.5–6.3) suggests better bone penetration than vancomycin (ratio of bone-to-serum concentration: 0.27) (Rao et al., 2011). However, the rapid antibiotic resistance evolution of the bacteria has become one of the most frequently stated problems in antimicrobial therapy. The increased rate of multi-drug resistant infection in chronic osteomyelitis was observed in literature (Dudareva et al., 2019; Wu et al., 2019).

The surgical considerations include radical sequestrectomy, dead space management, soft tissue reconstruction and restoration of bone stability. Surgical removal of the sequestrum is paramount to a successful treatment outcome because sequestrum performs as the permanent source of virulent pathogens. All devitalized tissues need to be removed, with a wide resection margin of 3–5 mm and the establishment of adequate blood flow for effective systemic antimicrobial therapy (Lima et al., 2014; Barakat et al., 2019). However, the spatial heterogeneity of bacterial colonization in the bone and the surrounding tissue makes it impossible to ensure the complete eradication of bacteria despite of extended resection. Proper management of dead space is an important complement to kill the residual bacteria. Antibiotic-impregnated bone grafts would elute bactericidal levels of antibiotics for prolonged periods of time with minimized side effect. After the infection is controlled, soft tissue closure and vascularization are carried out to create a suitable environment for the following bone restoration. Autogenous bone implantation is usually applied for restoration of bone stability. Segmental defects longer than 3 or 4 cm require the Ilizarov technique or a vascularized pedicled bone graft (Khira et al., 2013). For smaller defects, autologous cancellous bone graft is sufficient. Finally, the treatment aims to rehabilitate patients to full weight-bearing. However, palliative treatment would be undertaken for infection remission and pain relief if the patient could not undergo such complex interventions. Additionally, we would foresee the periodic exacerbations of chronic osteomyelitis under the palliative treatment.

## 4 Classification and evaluation of biomaterials for osteomyelitis treatment

**FIGURE 1:**
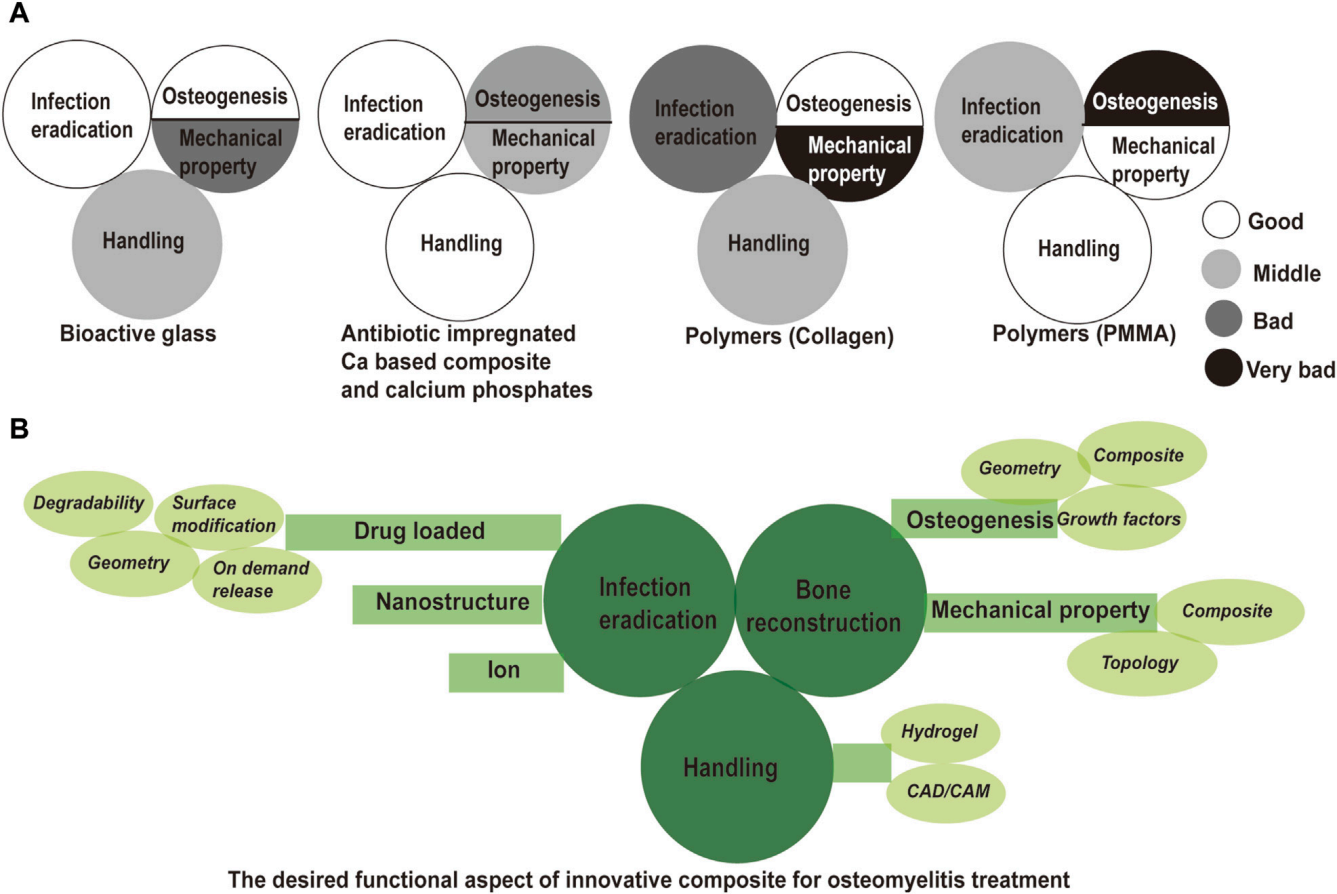
Evaluation of commercial bone void filler and solution for innovative composites in osteomyelitis treatment. **(A)** Evaluation of commercial bone void filler from infection eradication, bone reconstruction and handling; **(B)** solutions in innovative composite for a successful treatment outcome of osteomyelitis. Drug loaded, nanostructure, and ion modification are described for infection eradication. Surface modification, tunable degradability, tunable architecture and on demand release are introduced for controllable drug release. Enhanced osteogenesis and enhanced mechanical property are required for subsequent bone reconstruction. Introduction of growth factor, optimized architecture, and designed hybrid are indicated to achieve good bone regeneration and mechanical support. Hydrogel and CAD/CAM could be implemented for good handling property.

The combination of a degradable bone substitute with a localized antibiotic release device may achieve eradication of the infection and promote bone regeneration concurrently. This strategy is an appealing option for the management of dead space and bone restoration by one-stage surgery, thereby reducing the cost and shortening hospital stays (Pincher et al., 2019). If not feasible, the bone substitute would help osseous repair and complement subsequent autologous bone implantation in a compromised condition. There is a wide range of synthetic materials for the treatment of osteomyelitis on the market, such as bioactive glasses, calcium sulfate hemihydrate, and calcium phosphates, and polymers. We would introduce these materials from infection eradication, bone reconstruction (osteogenesis and mechanical property), and material-caused complication (see Figure 1).

### 4.1 Bioactive glass

Bioactive glass has intrinsic antimicrobial, osteoconductive, and angiogenic properties. Hydroxyapatite (HA) layers can form on the surface of the material and create an intimate bond with the bone tissue. Bioactive glass in fluid leads to the release of ions, an increase in osmotic pressure, and pH. In the process, “needle-like” sharp glass debris forms on the surface of bioactive glass. This structure creates hollows and holes on the cell wall, and damages bacterial membrane (El-Rashidy et al., 2017). Neither development of resistance under bioactive glass nor biofilm formation on the surface have been observed to date (Coraça-Huber et al., 2014). A 90% success rate confirmed BAG-S53P4 as a bone substitute in the treatment of chronic osteomyelitis with excellent results, irrespective of the isolated pathogen and the host condition (Romanò et al., 2014; Lindfors et al., 2016). However, Nina et al. reported that infection at less than 6 months post-operation in polymicrobial cases were significant more often observed than the one with no infection or single bacterial specie infection (Lindfors et al., 2016). Bone defects with a maximum size of 30–60 cm^3^ were filled by bioactive glass in the lower extremities, which indicated enough load-bearing capacity of the granules in a well-confined environment. Bioactive glass filling is not appropriate for segmental bone defect. In addition, handling property of bioactive glass is fair, as the granules migrate during and after surgery. A therapeutic error was reported due to inadequate filling of these granules in a long-nail defect crossing both the tibia and femur (Al Malat et al., 2018). Bioactive glass degrades slowly and will remain in body for several years. Seroma leakage is thought to be an inflammatory response after implanting the material in the early stages of healing. It is presumed to be the result of osmotic effects caused by the material. The reported complication rate of seroma leakage after bioactive glass implantation was 2.6%–3.7% (Drago et al., 2013; Lindfors et al., 2016).

### 4.2 Antibiotic-impregnated calcium-based bone substitutes and calcium phosphates

There are several calcium-based bone substitutes and calcium phosphates with chemical and crystal structures somewhat similar to the inorganic composite of bone, such as calcium sulfate. Such devices provide a good infection control rate varies from 76.7% to 100% (McKee et al., 2002; McKee et al., 2010; Romanò et al., 2014; Niikura et al., 2016; Karr, 2018; Qin et al., 2018; Visani et al., 2018; Badie and Arafa, 2019; Qin et al., 2019; Qin et al., 2020). The reinfection rate was reported to be limited to 3%–13% (Visani et al., 2018; Qin et al., 2019; Qin et al., 2020). Calcium sulfate degrades very fast, which has been reported to cause early resorption of the material in 1 month (McKee et al., 2002). The osteoconductive effect is hampered after quick hydrolysis of calcium sulfate as the scaffold needed for bone regrowth disappears. Only partial ingrowth of the bone into the void defect was observed at 6 months post-operation. Seroma leakage rate was reported to be around 30%, higher than that of bioactive glass (Qin et al., 2019). Postoperative material leakage was common when calcium sulfate was implanted, because: i) there was insufficient soft tissue to cover the material when the lesion was too superficial; ii) calcium sulphate was squeezed out of the cavity during the healing process. Although the complication does not lead to reinfection, it may distress the patients as the substance leaking out of their wounds. Transient hypercalcemia has also been frequently reported as an acceptable complication. Calcium phosphates attract increased attention because their degradation products can be used for new bone formation. Calcium and phosphate ions are indeed known to regulate bone metabolism (Habraken et al., 2016). HA is a calcium phosphate. By combining calcium sulfate with HA, biocompatibility is increased, and inflammatory responses are reduced. HA compensates for early resorption of calcium sulfate and provides long lasting scaffold for bone repair. Signs of minor extraosseous leakage of these materials into the surrounding soft tissues, which were visible on the radiograph, were reported to be 11.7% (McNally et al., 2016). Calcium phosphate cement (CPC) would reach a compressive strength as high as 80 MPa in 24 h after implantation, which can be applied for remedy of bone defects and support of fixation for the fractures requiring reduction (Lim et al., 2002). However, CPC degrades slowly and remains in body for several years. If the recurrence of infection becomes evident, the removal of CPC should be planned. In one report, CPC was implanted in a 2 cm segmental defect with external fixation, and then changed into an autologous bone graft, indicating an acceptable mechanical strength and limited osteogenesis ability (Niikura et al., 2016). After mixing the powder and liquid, these commercial bone void fillers can form moldable pastes with good handling. These pastes can be injected into closed bone defects, and self-setting to obtain mechanical stability.

### 4.3 Polymers

Polymers have been divided into natural polymers and synthetic polymers. The natural polymers, such as collagen, chitosan, and gelatin, are always hydrophobic and highly biocompatible. The major drawback of using natural polymer in composites is their tendency to arouse the immune response *in vivo*. Topical gentamicin–collagen sponge has been applied for diabetic patients with foot ulcer infection (Elumalai et al., 2018; Uçkay et al., 2018). A systematic review reported that the eradication ratio of infections in the treatment of chronic osteomyelitis using gentamicin collagen sponge ranged from 63% to 100% (van Vugt et al., 2018). The efficacy of these antibiotic-loaded collagen sponges in orthopedic surgery is restricted due to their limited mechanical property. It is more frequently applied in patients with soft-tissue-related infections. The safety concerns of high concentration antibiotics, owing to disadvantages regarding pharmacokinetics (released completely within mostly 3–4 days), remain inconclusive and may lead to the development of renal failure (Hayes et al., 2016).

PMMA is a type of synthetic polymer, which is easy to use with good mechanical strength for osteomyelitis treatment. After gentamicin–PMMA beads implantation, infection eradication was achieved in 80–100% of patients (Barth et al., 2011). However, it is non-degradable and needs to be surgically removed in the treatment of osteomyelitis. The other degradable synthetic polymers such as poly (ε-caprolactone) (PCL) and poly (lactic acid-co-glycolic acid) (PLGA) have been widely utilized as cell-supporting matrices for bone repair (Wei et al., 2018; Qian et al., 2019). The physicochemical properties of polymers can be adjusted to fit the application of clinical therapy such as longer shelf life, uniformity of microstructure, high mechanical strength, and reproducibility. But hydrolysis of degradable polymers as PLGA causes a drop in local PH, which may lead to foreign-body responses and bone resorption (Li et al., 2011). The blend of polymers is advantageous. PCL is hydrophobic and degrades slowly, whereas PLGA is relatively hydrophilic and degrades quickly with high water-uptake ability. PCL and PLGA were melt in high temperature and blended with tobramycin powders, which was introduced as a printable material. 3D-printed PCL/PLGA scaffold was observed with a 2-month release profile of tobramycin, and the *in vivo* efficacy of scaffolds for the treatment of chronic osteomyelitis demonstrated low inflammatory responses and remarkable ingrowth of new bone formation in a rat model (Toiyama et al., 2015). The synthetic degradable polymeric bone substitutes have been introduced in experimental osteomyelitis in literature, but continued efforts are needed to make them more accessible to clinical treatment.

## 5 Systematic review of the literature on the antimicrobial biodegradable bone substitute as a part of osteomyelitis treatment

### 5.1 Search strategy

All types of prospective and retrospective cohort studies concerning the clinical application of different synthetic bone substitutes in the treatment of osteomyelitis for adults were assessed. A bibliographic retrieval was carried out through searching articles in PubMed, Scopus, and Web of Science until 2023. Search terms included “osteomyelitis,” “anti-bacterial agents,” “bone substitute” and “clinical trial.” English language and human species restriction were chosen. Consequently, the search strategies initially provided a total of 180 records in the PubMed database. These results were evaluated on their titles and abstracts, resulting in 162 studies to be discarded because of different reasons: 99 reports were irrelevant; 16 reports were review type; 17 reports lacked a biodegradable synthetic graft; 7 reports were specifically on the treatment of diabetic foot osteomyelitis, which was a different disease compared with others; 7 with limited patient number for evaluation; 6 reports aimed at different aspects but no treatment outcome; 10 reports were case reports. Based on retrieval and analysis of literature in the above databases, 18 articles finally meet the assessing standards.

### 5.2 Results

As can be seen in Table 1, a total of 18 reports concerning degradable antimicrobial bone substitutes for the management of dead space in the treatment of osteomyelitis, 12 retrospective reports, and 6 prospective reports were included for analysis. Among these, 4 reports involved bioactive glass, 8 reports involved calcium sulfate, 4 involved HA calcium-sulfate compound, and 2 involved calcium phosphate cement. We focused on the eradication of infection initially as it is the primary treatment purpose. The outcome for infection eradication ranged from 50% to 100% for observation periods ranging from 9 to 95 months, as assessed by clinical manifestation, radiographic examination and/or laboratory examination within the observation period. Among these studies, Lindfors et al. have reported a multicentral RCT by comparing a one-stage procedure (bioactive glass group) with a two-stage procedure (PMMA group) for 15–95 months (Lindfors et al., 2016). Reinfection was significantly more often observed when PMMA was used for treatment. Michael et al. reported that the infection eradication rate in treatment with calcium sulfate showed the same proportion as the treatment with PMMA. However, the union formation in treatments with calcium sulfate was 100%, whereas that for treatment with PMMA was 87.5% (McKee et al., 2010). These two studies were both RCTs, which are much more optimal than the other included prospective and retrospective studies to assess the efficacy of the treatment of degradable antimicrobial bone substitutes. We concluded that biodegradable antimicrobial bone substitutes are robust management strategies for osteomyelitis treatment, compared with the two-step procedure using PMMA.

**TABLE 1:** Included studies for the treatment outcome analysis of chronic osteomyelitis.

Study	Patient	Treatment	Antimicrobial function	Trials	References	Volume of bone defect/cm^3^	Infection eradication (%)	Union/Bone formation	Complication	Follow up/m
1	98/18	Bioactive glass (BAG-S53P4)/PMMA followed with BAG-S53P4	Intrinsic ability	Prospective multinational RCT	Lindfors et al. (2016)	N/A	95%/67	N/A	Reinfection: 8%; non-unions; pulmonary embolism; pain syndrome; fracture	15–95
2	27	Bioactive glass (BAG-S53P4)	Intrinsic ability	Prospective study	Drago et al. (2013)	21.0 ± 10.9 (2–60)	88.9	N/A	Reinfection: 7.41%; serum leakage:3.7%; delayed skin necrosis	9–30
3	50	Bioactive glass (BAG-S53P4)	Intrinsic ability	Retrospective study	Al Malat et al. (2018)	11.1 ± 6.7 (3–30)	86	83.3%	Reinfection: 14%; chronic pain syndrome	2–27
4	27/27/22	Bioactive glass (BAG-S53P4)/HA and calcium sulphate compound/Mixture of tricalcium phosphate and demineralized bone matrix	Vancomycin and meropenem and teicoplanin	Retrospective study	Romanò et al. (2014)	N/A	92.6%/88.9%/86.3	N/A	Serum leakage: 3.7%/29.6%/27.2%; deep vein thrombosis; fracture; delayed skin necrosis and bone exposure; renal dysfunction	12–36
5	25	Calcium sulfate	Tobramycin	Prospective study	McKee et al. (2002)	30.5 (3–192)	92	Bone formation:100%; Nonunion to unite: 87.5%	Reinfection: 8%; serum leakage: 32%; nonunion; refracture; skin necrosis	20–38
6	25/40	Calcium sulfate/Debridement	Tobramycin	Retrospective study	Chang et al. (2007)	29.2 (10–80)	80%/60	N/A	Reinfection: 20%/40%	36–334
7	15/15	Calcium sulfate/PMMA	Tobramycin	Prospective RCT	McKee et al. (2010)	37.5 (3.1–120)/27.5 (4.4–126)	86%/86	Bony defect repair: 100%/100%; nonunion to unite: 100%/87.5%	Reinfection; refracture	24–60
8	54/20	Calcium sulfate/Wound irrigation-suction	Vancomycin	Retrospective comparative-cohort study	Qin et al. (2019)	86.11 mm (76.72–95.51)/82.25 mm (66.17–98.33)	90.74%/45	N/A	Reinfection: 1.85%/25%; docking site obstruction: 1.8%/25%; material postoperative leakage:35.2%	28.5/30.5
9	354	Calcium sulfate	Vancomycin and gentamicin	Retrospective study	Gauland (2011)	5–20	93.8	N/A	Serum leakage	60
10	30	Calcium sulfate and bone marrow aspirate added to the bio-composite	Vancomycin and gentamicin	Prospective study	Badie and Arafa (2019)	N/A	76.7	83.3%	Reinfection: 16.7%; serum leakage: 50%; refracture	≥12
11	38/43	Calcium sulfate	Vancomycin	Retrospective study	Qin et al. (2020)	28.3 (5–50)	88.4	N/A	Reinfection: 11.6%; serum leakage: 30%; slight pain after a long-distance walk:10.5%; limb weakness or discomfort: 7.9%; fibrous scar formation: 5.2%; joint stiffness: 2.6%; slight claudication: 2.6%	35.9–89.9
12	34	Calcium sulfate	Vancomycin and gentamicin	Retrospective study	Jiang et al. (2020)	N/A	85.29	N/A	Wound leakage	12–68
13	52/45	HAs calcium-sulfate/Gelatin sponge or Calcium sulfate or PMMA or nothing	Vancomycin or gentamicin or imipenem	Retrospective study	Visani et al. (2018)	N/A	86.5%/100%, 50%, 50%, 80	N/A	N/A	≥24
14	125	HA calcium sulfate with percutaneous antibiotic delivery technique	Vancomycin	Retrospective study	Karr (2018)	2–3	96.15	N/A	Reinfection: 3.85%	74
15	13	Calcium phosphate cement	Vancomycin or gentamicin or arbekacin	Retrospective study	Niikura et al. (2016)	N/A	100	N/A	N/A	50.4 (37–73)
16	49/49	Calcium phosphate cement/Irrigation and drainage device	Vancomycin	Prospective RCT	Lang et al. (2021)	N/A	93.8%/73.4	61.2%/32.65%	N/A	12
17	21/10	Calcium sulfate-calcium phosphate composite/Calcium sulfate	Vancomycin	Retrospective study	Zhao et al. (2020)	5.28/5.66 cm (length); 2.31/2.28 cm (width)	100	20.5%/15.4%, 43.7%/32.2%, 75.2%/49.7% at 1, 3, and 6 months	Reinfection: 0/20%; delayed wound healing:4.1%/30%	15.3/21.7
18	163	HA calcium sulfate compound	Gentamycin sulfate	Retrospective study	Ferguson et al. (2019)	10.9 (1–30)	95.7	73.8%	Infection recurrence: 4.3%; material extraosseus leak: 15.95%; overall fracture: 2.5%	21.4 (12–56)

By comparing different materials, the eradication of infection ranged from 86% to 95% when using bioactive glass, from 50% to 93.8% when using calcium sulfate, and from 85.6% to 96.15% when using HA/calcium sulfate. Calcium phosphate cement showed a 100% infection eradication in 13 patients in one study (Niikura et al., 2016). Some reports directly compared different commercial void fillers in the retrospective analysis. HA calcium sulfate compound, compared with a mixture of tricalcium phosphate and demineralized bone matrix, bioactive glass has been reported to have a higher cure rate of 92.6%–88.9%, 86.3% but not significant, respectively (Romanò et al., 2014). In the other study, the healing rates were as follows: 86.5% in treatment with HA calcium sulfate compound, 100% in treatment with gelatin sponge, 50% in treatment with calcium sulfate, 50% in treatment with PMMA, and 80% in treatment with only debridement. The healing rate of HA calcium sulfate was significantly higher (Visani et al., 2018). However, these two studies may have introduced some selection bias into the results. Therefore, it is difficult to decide what materials is better. The selection of these commercial bone void fillers should be comprehensively considered including the local conditions, systemic factors, and the cost, etc.

Osseous repair occurs after the infection is eradicated, which is influenced by the degradation of the implanted material. The degradable product of bone substitute as calcium ions has a profound effect on osteoblast proliferation (Chai et al., 2012). Theoretically, the process of material resorption and the replacement of the created void with new bone should occur simultaneously and at a gradual pace. In our literature review, we identified four studies that examined the ratio of patients who achieved new bone formation and those who experienced nonunion, as determined through radiological evaluation. The union/bone formation rates ranged from 50% to 100% across these studies. Specifically, one study reported a union/bone formation rate of 83.3% in the treatment of bioactive glass and 83.3%–100% in the treatment of calcium sulfate. However, it is important to note that the criteria for evaluation of union/bone formation is inconsistent. One study assessed osseous consolidation in the bony defect with the treatment of bioactive glass through radiographic examination (Al Malat et al., 2018), while the others calculated the percentage of new bone formation into the bone void with the treatment of calcium sulfate (McKee et al., 2002; McKee et al., 2010; Badie and Arafa, 2019). Among these, one study set 60% or greater as the evaluation standard (McKee et al., 2002). Additionally, two studies reported the ratio of nonunion to unite (McKee et al., 2002; McKee et al., 2010), and the healing of nonunion was defined as bridging of three out of four cortices in one study (McKee et al., 2010). This inconsistency may be partially attributed to variations in material degradation. These materials serve as osteoconductive matrixes to induce osseous repair. Bioactive glass remained visible in radiography 1 year after implantation, while calcium sulfate disappeared 3 months after debridement and implantation. It is crucial to control the material resorption rate to ensure proper load transfer to the developing bone. Connective tissue would grow into the bone defect to form biomechanical inferior structures after early resorption of implanted material. Transient loss of mechanical support increases the risk of postoperative fractures, as Table 1 shows refracture to be a common complication. In contrast, the alloplastic material with slow degradation would exist *in vivo* for a long time and should have excellent biocompatibility, making it unable to provoke a foreign body effect.

Bone defect volume affects the bone healing process. In the 10 studies displayed in Table 1, the volume of bone defects ranged from 2 to 60 cm^3^ in the treatment with bioactive glass, and from 3 to 192 cm^3^ in the treatment with calcium sulfate. Small bony defects are usually structurally stable, which is easy to treat when there are no systemic or local compromising factors. An atraumatic percutaneous antibiotic delivery technique was successfully introduced to inject the HA calcium sulfate paste into 2–3 cm^3^ bone defects with a simple procedure (Karr, 2018). However, larger bony defects require a more complex and difficult procedure to obtain bone stability and viable vascularized tissue. Serial grafting for larger defects is required occasionally as initial antimicrobial graft, soft tissue transplant and final autologous bone graft. These synthetic bone substitutes are unable to stimuli adequate bone regeneration in a compromised large defect. Addition of bone marrow aspirate might partially compensate for the limited osteogenic properties of the bone substitute but it is still inadequate for large bone defects (Badie and Arafa, 2019). The use of bone morphogenetic proteins (BMPs) may enhance the bone substitute’s osteoinductive ability for bone reconstruction (Hsu et al., 2016). More strategies will be explored to facilitate future bone substitutes with excellent infection eradication and osseous repair abilities.

## 6 Drug incorporation and antibiotic options

These bone substitutes are suggested to provide adequate, sustained, and controllable presentation of drugs in a time-dependent manner. The efficacy of antibiotics in killing bacteria may be influenced by the peak concentration surpassing the breakpoint (concentration-dependent activity) or by the duration of time during which concentrations remain above the breakpoint minimum inhibitory concentration (MIC) (time-dependent activity) (Yılmaz and Özcengiz, 2017). Killing bacteria in the sessile form with slow metabolic rate requires much higher concentration of antibiotics than the planktonic form with high metabolic rate. Hence, an optimal drug release profile for anti-infection should be a constant release with local high concentration for a long duration. The local high concentration of drug should not affect the process of bone healing, either. Considering the global issue of antibiotic resistance, drug release profile should be tailored to the requirements of individual patients. However, drug release profile usually showed initial burst release and the plateau stage in most bone substitutes. The high initial drug release reduces the effective lifetime of the device. The low antibiotic release in the plateau may increase the risk of antibiotic resistance.

Antibiotics are impregnated into commercial bone void fillers by simply blending drugs with the synthetic bone substitutes. The antibiotics should cover a broad spectrum of bacterial pathogens involved in chronic osteomyelitis. Some combinations of antibiotics, such as vancomycin and gentamicin, were applied to be active against both Gram-positive and Gram-negative bacteria (Gauland, 2011). The drugs are mainly hydrophilic, whose release in such systems primarily depends on the dissolution rate of the drug from its matrix (Nandi et al., 2009). Antibiotics should be successfully mixed with the powders to form fully hardened cements. After self-setting, the antibiotics should be able to elute from the matrix. After being eluted, they should still maintain efficacy against the pathogenic microorganisms (Verron et al., 2010).

Experimentally, antibiotics are entrapped in microspheres, nanoparticles, nanotubes, 3D-printed scaffolds, and electrospun fibers (Toiyama et al., 2015; Hassani Besheli et al., 2017; Topsakal et al., 2018; Aksoy et al., 2019). The processing procedures, including double emulsion-solvent extraction, lyophilization, inkjet printing, chemical crosslinking, and electrospinning, might be harmful to the antimicrobial efficiency of antibiotics. Organic solvents and inorganic compounds are used, which might break the structure of antibiotics and lead to their inactivation. Antibiotics might undergo high temperatures when mixed with molten polymers (Toiyama et al., 2015). Besides, antibiotic should be resistant to the degradable products of the experimental material. Most frequently investigated materials such as PLGA produce acidic substances that may accumulate and reach high concentrations (Li et al., 2011). Biocatalytic-based materials are decomposed enzymatically, hence antibiotics should be able to withstand these enzymes (Park et al., 2007). Drugs such as vancomycin, tobramycin, and gentamicin, should maintain stability during both loading process and subsequent release period of the experimental materials, which possess chemical, physical, and biological resistance.

Antibiotics in commercial bone void fillers for the treatment of osteomyelitis are mainly vancomycin, gentamicin, tobramycin, meropenem, and imipenem according to the prescribed dosage. Vancomycin is s a glycopeptide active against Gram-positive bacteria including *S. aureus*, considered for most cases of osteomyelitis. Tobramycin and gentamicin are aminoglycosides with an aerobic Gram-negative bacilli cover as well as *S. aureus*. Meropenem and imipenem are broad-spectrum β-lactam antibiotics against both Gram-positive and Gram-negative bacteria. These drugs have excellent elution properties *in vitro* from various carriers. Finally, the choice of antibiotics in local carriers should be not only empirical but also based on a definite microbiological diagnosis and a personalized condition. Renal function should be closely monitored in patients with known or suspected renal impairment after implantation of tobramycin loaded calcium sulfate during osteomyelitis treatment. This would generate an alarming high rate of high serum concentration of aminoglycoside based on the instruction of Osteoset-T.

Antibiotic resistance remains a major challenge for chronic osteomyelitis treatment. More and more antibiotic-resistant bacteria have been reported, mainly due to pathogens variants with biofilm formation, altered metabolic activity, and acquired genetic mutation (Cobb et al., 2020). Biofilm formation prevents the antibiotics into the avascular region and protects the inner bacteria from antibiotics. Poor drug penetrability prevents the attainment of required MIC levels in bone tissue, prolonging the course of the therapy and leading to antibiotic resistance. Moreover, the sessile form of pathogens reduces the sensitivity to antibiotics compared with the planktonic phase. For example, *S. aureus* can develop an altered bacterial phenotype with a very slow metabolic rate, referred to as small colony variant, which are more resistant to antibiotics and may be more apt to form biofilms (Tuchscherr et al., 2016). When bacterium invade the canalicular networks of cortical bone, they achieve protection from the immune system and cause a slow, persistent, and indolent infection. The osteocyte lacuna-canalicular network is an ideal architecture for *S. aureus* to attach, form biofilm, and hide from immune attack (Masters et al., 2019). Thus, in order to reduce antibiotic resistance and achieve the high-efficiency antimicrobial goal, more approaches for drug assembly, modification, delivery, and release are needed to be further studied.

## 7 Commercially available bone void fillers and their characteristics

It is usually preferred for the treatment of established infections that high doses of antibiotics in either liquid or powder phase simply blend into local carriers, whose handling property is excellent. Table 2 shows the commercial bone void fillers and their characteristics of drug release, and degradation, based on instructions and literature. The recommended dosage of drugs is mainly based on the bactericidal power of drugs and their release rates from different systems. Overall, elution characteristics of drugs show that the burst release (highest concentration) occurred at the initial 24–48 h, declined slowly, and continued to maintain therapeutic levels for a long period of 10–28 days (Rauschmann et al., 2005; Turner et al., 2005; Stravinskas et al., 2016). Their degradability should be considered in combination with clinical demand. Different materials chosen depends on the patient’s situation and the types of bone defects. For instance, bone granules are more suitable for tiny bone defects than pellet. Injectable bone substitutes are more suitable for irregularly shaped bone defects than other types. However, there are still some complications related with those bone void fillers, such as serum leakage, fracture, delayed skin necrosis, etc. A hydrogel could be combined with granular bone substitute to obtain a mouldable/injectable synthetic bone substitute (Pereira et al., 2019). This combination can serve as space-holders to prevent granule packing and allowing the clinicians to handle and shape the formulations into the bone defects without leakage. Overall, more techniques are needed further studied to create optimal bone substitute materials.

**TABLE 2:** Summary of commercially available bone void fillers.

Manufacturer	Product name	Carrier	Antibiotics	Delivery	Drug phase and dosage	Antibiotic release	Degradability
BonAlive Biomaterials	Bioglass	BAG-S53P4	-	Granules	-	-	Degrade slowly and exist in body for several years; observed thickening of the neocortex
WRIGHT medical group	Osteoset-T	Calcium sulfate	Tobramycin	Pellet	Powders; 4% (weight%)	Over 10 days	Early fully resorption in 3–6 months, 64% bone growth into the bony void
Biocomposites	Stimulan Rapid Cure	Calcium sulfate	Vancomycin	Pellet/Paste	Powders; 10% (weight%)	Over 40 days	Early fully resorption; 50% patient achieved full bone regrowth
			Gentamicin		Liquid; 40 mg/mL		
			Tobramycin		Liquid; 40 mg/mL		
aap Biomaterials GmbH	PerOssal	HA/Calcium sulphate compound	Gentamicin	Pellet	Liquid; 40 mg/cc	Over 10 days	6m–18 m Fully resorption
			Tobramycin		Liquid; 40 mg/cc		
			Vancomycin		Liquid; 50 mg/cc		
			Rifampicin		Liquid; 60 mg/cc		
Bonesupport	CERAMENT G	HA/Calcium sulfate compound	Gentamicin	Paste	Liquid; 17.5mg/cc	Over 28 days	Fully resorption after 11 m
Bonesupport	CERAMENT V	HA/Calcium sulfate compound	Vancomycin	Paste	Liquid; 66mg/cc	Over 28 days	Fully resorption after 11 m
Hoya Pentax	Biopex-R	Calcium phosphate cement	Vancomycin	Paste	Powders; 4%/8% (weight%)	Over 36 weeks	Non degradable
			Gentamicin		Liquid; 0.5% (weight%)		
			Arbekacin		Liquid; 0.8% (weight%)		

Innovative composites were constituted of bioactive glass, calcium-based composite, calcium phosphates, and polymers. Drug loaded, nanostructure, and ion modification endows the composites with antimicrobial capacity to eradicate infection in osteomyelitis treatment. Drug loaded composites could accomplish controlled drug release but may produce harmful effects as initial high concentration and induction of bacteria resistance. Since drug release were affected by drug diffusion and material degradability, controlled drug release would be achieved by surface modification, tunable degradability, and tunable architecture. Surface modification would limit the initial burst release of drug. On demand release and antimicrobial peptide might reduce the risk of antibiotic resistance. Ion modification would produce some ion as Zn^+^, and endow the material with intrinsic antimicrobial ability. These ions could induce cell proliferation and cell differentiation for subsequent bone healing. However, the antibacterial efficiency of ion modification and its cytotoxic is doubtful. Nanostructure modification would enhance bacteria killing efficiency, kill intracellular bacteria, overcome bacteria resistance, and kill the bacteria in the biofilm. Mesoporous bioactive glass would provide a porous structure and large surface area for drug loading.

## 8 Advanced composite biomaterials for osteomyelitis treatment from experimental work

**FIGURE 2:**
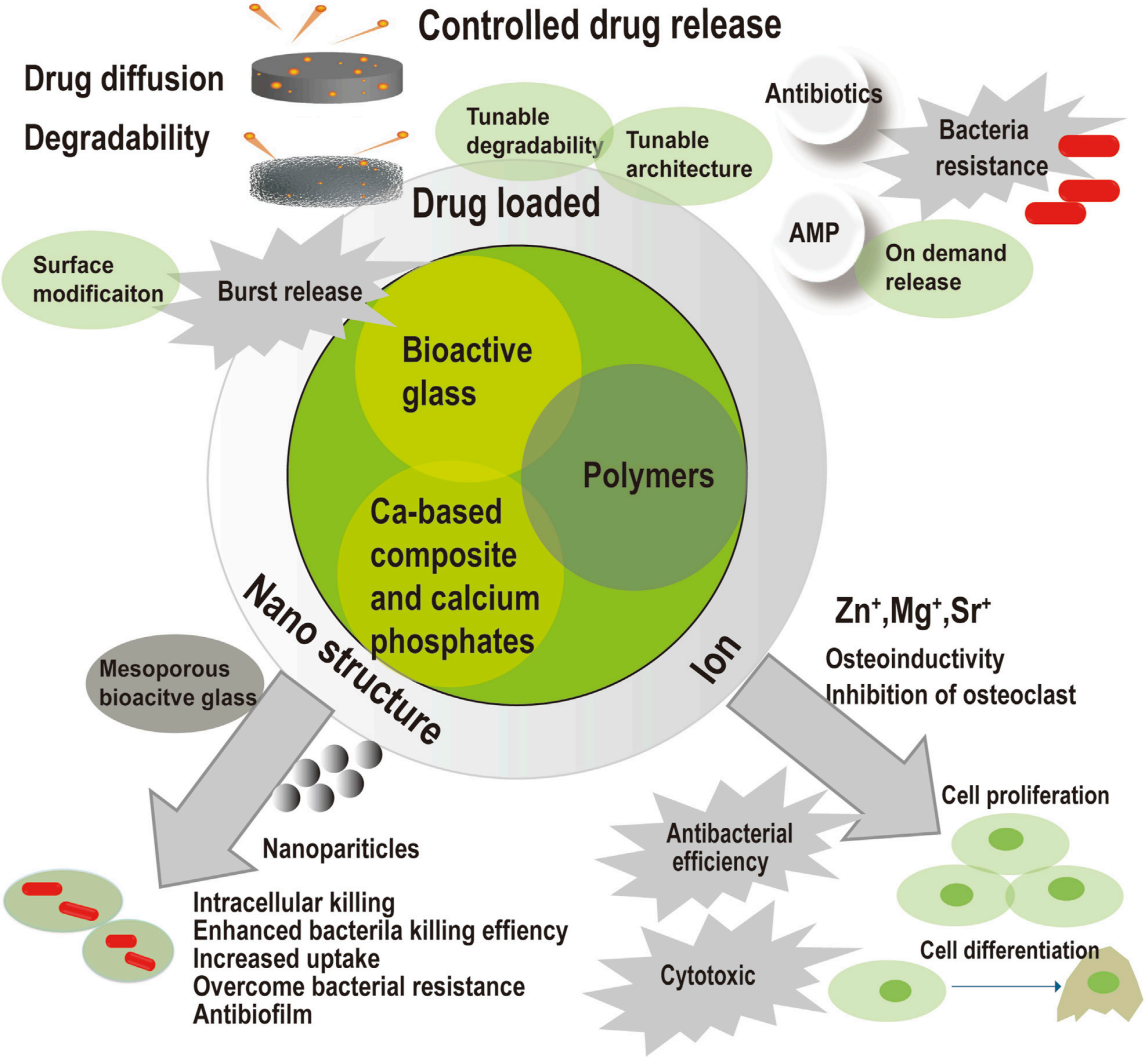
Infection eradication of innovative composites in the treatment of osteomyelitis.

The commercially available bone void fillers in the table perform well in the treatment of osteomyelitis. However, there are still some limitations, including slow degradation, low mechanical strength, inadequate infection eradication and bone regeneration stimulation, and a high resorption rate. A bibliographic retrieval was carried out through searching articles in PubMed, until 2023. Search terms included “antimicrobial,” “degradable,” and “bone substitute.” Represented references were chosen and divided into 3 parts, including infection eradication, bone reconstruction and handling property based on advanced and promising strategies and good results. As shown in Figure 2, many strategies have been involved to improve the conventional antimicrobial bone substitutes in terms of infection eradication, bone reconstruction, and handling. Organic-inorganic hybrids and composite biomaterials with hierarchical porous structures and advanced processing techniques have been introduced for better outcome in bone healing and function.

### 8.1 New strategies for infection eradication

The foremost purpose of osteomyelitis therapy is infection eradication. New strategies may achieve the prospective high-efficiency antimicrobial goal of providing a sterile bed for bone regrowth.

#### 8.1.1 New strategies for manipulating drug function

Antimicrobial drugs were incorporated into bone substitutes, endowing them with antimicrobial abilities. Manipulating the drug release profile would raise antimicrobial efficiency and increase the opportunities of infection eradication by tunable architecture, surface modification (electrostatic or covalent bond between drug and host matrix), tunable degradability, stimulus response release design, etc .,(Pawar and Srivastava, 2019; Tihan et al., 2019).

Sample geometry including surface area, pore volume, and pore number influences drug release rate from these bone substitutes. Nanotubes, liposomes, and microspheres as reservoir systems can provide a safe and efficient transfer by drug inclusion (Shutava et al., 2014). Mesoporous bioactive glass has a highly ordered mesopore channel structure with a pore size below 20 nm ang a large surface area. Mesoporous bioactive glass possesses excellent delivery ability by the encapsulation of pharmaceutical therapeutics as a reservoir device, and provides a well-interconnected pore structure where host cells can attach, spread, and proliferate (Cheng et al., 2018; Anand et al., 2019). Nanoparticles are expected to exhibit efficacy in killing intracellular pathogens, combating persistent infections (such as methicillin-resistant *Staphylococcus aureus*, MRSA), and demonstrating remarkable antibiofilm properties (Brennan et al., 2015; Mu et al., 2016). Smaller sized nanoparticles were suggested to possess enhanced capability to penetrate the bacterial cell membrane. Such nanoparticles could potentially act synergistically with antibiotics to kill the antibiotic resistant bacteria (Wu et al., 2018).

Surface modification techniques, involving immobilization and entrapment of drugs on the surface of material through the degradable linkers, may prevent burst release and extend the release duration. Bioactive glass nanoparticles with amino-functionalized vancomycin were reported to show a 20% release in the early stage, while the one without functioned was around 45% within 6 h (Zarghami et al., 2020). Drug release profile is also affected by matrix degradation and drug diffusion together in a controlled manner (Wu et al., 2020). For example, PLGA incorporation has been showed to generate acid, which can corrode bone substitutes and release entrapped drugs from the matrix. The acid product of PLGA coated calcium sulfate/biphasic calcium phosphate composite eluted with vancomycin hydrochloride and tobramycin sulfate have been implanted in MRSA induced osteomyelitis rabbit model and proved therapeutic effects. The *in vitro* results showed that the percent drugs release of antibiotics in 2 weeks was 89.3% of vancomycin and 18% of tobramycin respectively. Whereas, cumulative release proportion of drugs from PMMA beads were only 25% and 11.5%, respectively (Mistry et al., 2016). Recent research showed that drug adsorption and release are dependent on the adsorbent material and the drug polarity/hydrophilicity, leading to different distinct modes of drug adsorption and release. Based on this property, a controlled drug delivery system (activated carbon fiber cloth-aspirin/biomimetic apatite-tetracycline) was synthetized. This double adsorption functionality biomaterial has better capacity to inhibit infection and promote osteogenesis (Olivier et al., 2021).

Antibiotic resistance, which is becoming a major global public health crisis, leads to an estimated number of around 50,000 deaths annually in the United States and Europe (Morehead and Scarbrough, 2018). Drug assembly, modification, delivery, and release all have close correlation with antibiotic resistance. Drugs delivery and burst release were influenced by compacting methods. A calcium polyphosphate hydrogel (CPP) as matrix for delivery of vancomycin and erythromycin was prepared by mechanical compaction (at 3000 psipressure, C-discs) or by regular manual compaction (M-discs). Results showed a significant reduction of burst release of vancomycin and rythromycin in C-discs (1.8% and 5%) as compared to that from M-discs within 72 h (55% and 60%). In addition, C-discs significantly extended the vancomycin release (1,500 h) and rythromycin (800 h) as compared to M-discs (160 and 96 h). The vancomycin released from C-discs maintained its bactericidal activity much longer than that from M-discs (Chehreghanianzabi et al., 2022). The composites materials based on sodium alginate (ALG) cross-linked with nano-HA loaded with ciprofloxacin (CIP) showed favorable bioactivity and antibacterial properties. The results demonstrated the release of CIP was driven by ionic strength, which was controlled by ALG, reducing the burst release of drugs (Benedini et al., 2020). Our group has designed a tunable CPC/PLGA/carboxymethylcellulose (CMC) composite to deliver doxycycline. The drug release profile showed one stage liner-release and two stage Peppas-release, which was controllable predictable by a mathematic model. Hence, it might be introduced to reduce the antibiotic resistance in the therapy of osteomyelitis (Liu et al., 2023).

On-demand drug release would reduce the risk of antibiotic resistance and minimize the local toxic effects on surrounding bone tissues. According to the microenvironment around the infected area like acidic pH, elevated expression of inflammatory enzymes, excretion of bacterial specific toxin, generation of reactive oxygen species, etc., different stimuli-responsive polymer-antibiotic hybrid have been designed, which can selectively release the antibiotics at the infected area (Dey et al., 2021). For example, exotoxin can interact with the host cell plasma lipid membrane bilayer and disintegrate it by pore formation at the cell surface. By utilizing this pore formation ability, Zhang and co-workers built a bacterial toxin-responsive liposome formulation by stabilizing with chitosan-adapted gold nanoparticles (AuChi) to release vancomycin. In the infected area, the toxin secreted by *S. aureus* created pores at the liposomal surface and as a result bristly release of vancomycin can treat the infection selectively. When AuChi-liposomes treated with toxin, 40% pore formed on the liposomal surface within 1 h. Thus, these AuChi-stabilized liposomes could release 100% vancomycin within 24 h, when incubated with MRSA. But without bacteria, these liposomes restricted to deliver the antibiotic. The toxin-triggered locally released vancomycin was capable to inhibit the MRSA growth and treat the bacterial infection selectively and effectively (Pornpattananangkul et al., 2011). Moreover, a molecular gate was designed to enable mass transport control and respond to specific external stimuli. This molecular- or supramolecular-based system could be implemented in a porous scaffold for controllable drug release in osteomyelitis treatment. For example, mesoporous bioactive glass was functionalized with polyamines and capped with adenosine triphosphate. The molecular gate blocked the entrance of the mesopores and kept levofloxacin within the pore voids in the absence of bacteria. When bone infection exists, the activity of osteoclasts led to a notable rise in acid phosphatase levels, which correlated with a significant increase in adenosine triphosphate concentration. Subsequently, adenosine triphosphate was hydrolyzed by acid phosphate, thereby unblocking the surface of the pores and allowing for the release of the drug (Polo et al., 2018).

#### 8.1.2 Ion modification

Metal ions as Ag, Cu, Mg, Se, and Sr have been doped in inorganic bone substitutes, endowing their intrinsic antimicrobial ability and inhibition of biofilm formation, despite problems of host toxicity, or doubts about their efficacy. These metal ions injure bacteria by oxidative stress, protein dysfunction, or membrane damage (Martínez-Sanmiguel et al., 2019; Tovani et al., 2019; Uskoković et al., 2019; Yuan et al., 2020; Maqbool et al., 2021; Yang et al., 2022). Bivalent cations, such as Sr^2+^ and Mg^2+^, can substitute Ca^2+^ in the crystalline structure of inorganic biocomposites. The ion exchange dynamics between the ceramic and biological systems can potentially result in long-lasting antimicrobial properties and affect processes related to both bone formation and remodeling during the degradation of the ceramic material. Zn-doped sulfate-calcium-phosphate/cellulose nanocomposites has been proved to accelerate osteoblastic cell proliferation and mineralization by releasing trace amounts of Zn without causing an inflammatory response. Inhibition of bacterial growth was proved against *S. aureus* and *Escherichia coli* (Dharmalingam et al., 2019). Some studies have also confirmed the antibacterial, osteoinductive and anticancer activities of selenium substituted HA (Se-HA). Se-HA showed improved anticancer and antibacterial effects compared to pure HA. However, the limitation of use of selenium attributes to its potentially high cytotoxic effects. The addition of Sr^2+^ has been found to reduce the cytotoxic effects of selenium ions and to improve the cell proliferation and differentiation properties of HA (Uskoković et al., 2017; Maqbool et al., 2021). Another bone substitute composed of hydroxyapatite and MgO (HAp/MgO) has been proved to significantly reduce bacterial growth in comparison with pure HAp spherical granules. *In vivo* chicken embryo chorioallantoic membrane model showed the inclusion of MgO resulted in reduced inflammatory response and increased angiogenesis (Coelho et al., 2020). Similarly, surface-decorated graphene oxide sheets with copper nanoderivatives (GO/Cu) also showed excellent antimicrobial property. GO/Cu significantly inhibited the progress of bacterial infection and reduced the bacterial burden and inflammatory responses in a subcutaneous abscess model in rats. The major antibacterial mechanisms of GO/Cu were damaged bacterial membranes, promoted oxidative stress, and disordered crucial enzyme metabolisms (Yang et al., 2022).

However, these metal ions are considered not effective to eradicate established infections, but rather for prophylactic purpose to reduce the risk of infection. When implanted in the femurs of an osteomyelitis animal model, the result showed magnesium phosphate cement arrested bone destruction but did not heal the experimental osteomyelitis (comprehensively evaluated by infection control, bone integrity, and regeneration) (Mestres et al., 2019). Infection eradication involves high doses and long-standing antimicrobial agents, which may be ambiguous to bone regeneration. Infection eradication should not impair bone regeneration. Researchers should find a way out to equilibrate infection eradication and bone regeneration in the therapy of osteomyelitis.

### 8.2 New strategies for bone reconstruction

Bone reconstruction occurs after the infection is eradicated. Perfect bone reconstruction and remodeling facilitate bone integrity and weight-bearing. Otherwise, unsatisfied bone regeneration, bone nonunion, and insufficient bone reconstruction result in deformity of patients, which declines their quality of life. Bone substitutes implanted *in vivo* would provide a transient mechanical support and serve as a scaffold to induce formation of bone from surrounding tissues. Currently, one of the most promising strategies is to develop organic-inorganic hybrids or composite biomaterials, providing excellent possibilities for improving the conventional antimicrobial bone substitutes. The introduction of inorganic material into organic matrix has been investigated extensively as bio-mimics with the nature bone architecture.

#### 8.2.1 New strategies for enhanced osteogenesis

Osteogenesis occurs in bone reconstruction, which is a multi-dimensional process involving cells, osteoconductive matrix, osteoinductive signaling, and mechanical stability, etc. It is known that intrinsic properties of the materials have a profound effect on their mechanical and biological behavior (Martin et al., 2019; Pearson et al., 2019). For example, incorporation of collagen into calcium phosphates were extensively studied for bone regeneration, as collagen is a critical component of bone extracellular matrix and calcium phosphates show closest similarity to the mineral component of bone. Both collagen and calcium phosphates play important roles in osteoconductive characteristics (Inzana et al., 2014; Kozłowska and Sionkowska, 2015). The presence of interconnecting porosity is an important attribute of the osteoconductive matrix, as it supports the colonization of cells, continuous vascular ingrowth, the deposition of mineral matrix, and the transportation of therapeutic agents. Some growth factors participate in osteoinductive mechanisms, combined with the composite, to prolong the residence time of the protein and provide support for the invading osteoprogenitor cells. Therefore, a combination of these above elements was supposed to enhance osteogenesis. BCP granules are commercial calcium phosphate composites of β-TCP and HA with a porosity level of 80%. Initially, a porous multichannel BCP granule was coated with collagen, subsequently loaded with BMP-2, and then embedded into CPC with good handling. The *in vivo* study showed improved new bone formation and good degradability after implantation (Lee et al., 2017). For osteomyelitis, biodegradable sheath-core-structured drug-eluting PLGA nanofibers for sustainable and controllable delivery of antibiotics (vancomycin and ceftazidime) and rhBMP-2 via electrospinning have been designed. The sheath-core-structured nanofibers exhibited small fiber diameters and great pore size distributions with a string-like homogeneous rhBMP-2 distribution. The biodegradable coaxially nanofibers could release high concentration vancomycin/ceftazidime and rhBMP-2 for >4 weeks (Hsu et al., 2016). Then the researchers combined these nanofibers with a 3D-printed PCL scaffold, as PCL has a longer degradation time and preserves the osteoconductive properties of bone scaffolds during bone healing process. Then the scaffold was implanted into a critical segmental bone defect model in the femur of New Zealand rabbits. The results demonstrated satisfactory bone regeneration and excellent biomechanical reconstruction. A thick induced bioactive membrane was observed to form circumferentially around the applied scaffold, which was richly vascularized and would secrete growth factors to positively influence bone healing. The PLGA nanofibers had been dissolved completely, and only the PCL mesh was preserved after 8 weeks. The *in vivo* drug release showed local high concentration of antibiotics and BMP-2 for 42 days (Yu et al., 2020). Other researchers modified the chitosan-xanthan-based scaffolds chemically with introducing phosphorylated polymer (Chp) to their structure. The scaffolds have large pore sizes (850–1,097 μm), micro-roughness and thickness (0.7–3.5 mm in culture medium), as well as low thrombogenicity compared to standard implantable materials, extended degradation time and negligible cytotoxicity. More importantly, Chp-based formulations were capable to adsorb higher amounts of morphogenetic protein mimic (cytochrome C). When used *in vivo*, the material would be able to concentrate native BMPs and induce osteogenesis (Bombaldi de Souza et al., 2020).

It is now known that microenvironment change, induced by biomaterial-host immune responses and inflammation, plays an important role in bone reconstruction. Besides the capacity to directly stimulate osteogenic differentiation, the new generation of bone substitutes should be able to modulate the local immune microenvironment. A biomimetic baicalin-incorporating graphene oxide-demineralized bone matrix hybrid scaffold not only increased the osteogenic differentiation of BMSCs, but also promoted macrophage polarization towards the pro-healing M2 phenotype through the release of BAI. More importantly, the scaffold could promote significant bone regeneration in critical-size calvarial defects in rats (Guo et al., 2021).

However, to date, there has been no bone substitute that is equal to autogenous bone during the bone healing process. Presently, biomaterials primarily serve as osseous space holders and osteoconductive scaffolds. Ideally, the healing processes of bone reconstruction should result in perfect bone integrity and bone remolding without graft versus host reaction or residual foreign bodies.

#### 8.2.2 New strategies for enhanced mechanical properties

Bone provides structure and support for the body and enables mobility. The rehabilitation from osteomyelitis in some weight-bearing areas, such as tibia and femur, is difficult and essential, otherwise results in lameness. Therefore, the biofunctions of the biodegradable bone substitutes are stabilization of the coagulum, temporal load transmission, and stress distribution. Considerable mechanical strength is important for pain relief, either. The compressive strength of human cortical bone ranges between 90 and 230 MPa (with tensile strengths ranging from 90 to 190 MPa), whereas the compressive strength of cancellous bone ranges between 2 and 45 MPa.

Generally speaking, inorganic bone substitutes are stiff and brittle, making them difficult to shape. Composite biomaterials HA and nanobioglass ceramics can exhibit high compressive strength of ∼157 ± 2 MPa and high tensile strength of ∼83 ± 2 MPa(Algarni et al., 2019). Whereas, they are deficient in bending strength and fatigue. Organic substitutes are elastic as collagen. The inorganic-organic biocomposite hybrid would obtain the appropriate mechanical properties. Besides, condensation of the materials can enhance their mechanical properties while compromise their porosity. Although HA is bioactive and has high biocompatibility and osteoconductivity, it has poor mechanical proprieties including low strength, brittleness and low fracture strength. Carbon nanotubes (CNTs) are an excellent option to improve mechanical properties into composites due to its high aspect ratio, exceptional strength and stiffness. HA-reinforced with CNTs and bovine serum albumin (BSA) showed compressive strength in a range of 13–29 MPa (which is the range of trabecular bone) and more than 200% cell viability, which demonstrated their favourable cytocompatibility (Menezes et al., 2019).

Nanofibers and nanoparticle can achieve favorable mechanical properties and suitable porosity with optimal microstructure design. The researchers successfully synthesized highly efficient osteoconductive polymeric scaffolds with controllable pore size and mechanical strength. Various percentages of silver nanoparticle-decorated cellulose nanowhiskers were embedded into a chitosan and carboxymethylcellulose fabricated scaffold. These nanosized cellulose fibers, decorated with silver nanoparticles, were uniformly distributed in the polymeric matrix by hydrogen bonds formation. The inclusion of nanowhiskers led to an increase in both pore size and mechanical strength. The mechanical strength of the scaffold was adjusted from 0.35 MPa to 3.95 MPa, which is equivalent to cancellous bone (Hasan et al., 2018). Blend nanofibers (biocompatible dipeptide polyphosphazene-polyester blend) can be oriented in a concentric manner with an open central cavity to replicate bone marrow cavity, as well as the lamellar structure of bone (haversian canal), to build a 3D biomimetic hierarchical bone architecture scaffold. The compressive modulus of the scaffold was found to be 237 ± 30 MPa, which can keep shape at the maximum load of 500 N (Deng et al., 2011). Balancing function and regeneration are hypothesized to require both topology optimization and composite integration, which need further explorations.

### 8.3 New strategies for improved handling property

The bone substitutes range from porous block, granules, putty, and injectable cement, which are easy to use and cost-effective. Excellent handling property decides their commercial potentiality. The ideal synthetic bone grafts should be easily handled in clinically relevant periods and bind to bone well. In the process of surgery, surgeons can simply implant the graft and fill the bone defect without too much preparation. Then, the materials should effectively stabilize the defect without migration of material particles. A new class of highly tunable citrate-based fluorescent putty-like materials has been synthesized. Biodegradable photoluminescent polymers (BPLP) synthesized by reacting citric acid, 1,8-octanediol, and L-Serine (Ser) with HA, referred to as BPLP-Ser/HA putty. The material maintains soft mechanics and excellent handling because of the inclusion of citrate. BPLP-Ser/HA is malleable and can press-fit into irregular defects easily with comparable handling properties to bone wax. The osteopromotive effect of BPLP-Ser/HA was also evidenced in a cranial bone defect rat model (Tan et al., 2022).

Hydrogels are materials that swell with a substantial water content without dissolving in water, maintaining a distinct 3D network structure by crosslinks. The main advantages of the injectable materials are their minimally invasive application and their ability to fit irregularly shaped bone defects easily. Cross-linking of polymeric materials by metal ions and incorporation with nanoparticles are simple and effective approaches to obtain antimicrobial ability. The inclusion of these components enhances toughness and mechanical properties of hydrogel and even escalates cell adhesion (Tommasi et al., 2016; Xu et al., 2018). A dextrin-based hydrogel (HG) was combined with granular bone substitute to obtain a mouldable/injectable synthetic bone substitute. This combination can serve as space-holders to prevent granule packing and allowing the clinicians to handle and shape the formulations into the bone defects. When implanted in tibial defects in goats, the results showed that HG allowed the stabilization of the granules into the defect, ensuring effective handling and moulding properties of the formulation, as well as an efficient cohesion of the granules (Pereira et al., 2019). Recently, researchers created a therapeutic supramolecular assembly of chitosan and nanoclays for bone regeneration applications. These hydrogels could be prepared by a simple mixing procedure using laponite nanosheets (LNSs) and guanidinylated chitosan (GC). For clinical treatment, they can be prepared simply, and they have injectable and self-healing features that could facilitate, minimally invasive delivery to irregular defect sites and integration with surrounding tissues. Moreover, the hydrogel also showed intrinsic osteoinductive nature by activating the Wnt/β-catenin signaling pathway enhanced bone healing (Zhang et al., 2020).

3D-printed technics and custom-made computer-aided-design/computer-aided-manufacturing (CAD/CAM) technics facilitate and produce block scaffolds in the pre-setting of the desired anatomical form, which may extend the selection of substitutes and simplify surgical operation (Ding et al., 2013; Yu et al., 2020). The block graft is assumed to be prominent in critical-sized segmental bone defects due to its high mechanical strength. 3D printing is a promising technology that facilitates both spatial control over scaffold architecture to anatomically match complicated bone defect sites, and temporal control over the incorporated therapeutics to maximize their efficacy (van der Heide et al., 2022). For treating segmental bone defects in a rabbit femoral critical bone defect model, a 3D-printed PCL scaffold combined with coaxially electrospun PLGA/vancomycin/ceftazidime/BMP-2 nanofibers was developed. The material may facilitate bone healing by inducing bioactive membrane formation and yielding high concentrations of antibiotics and BMP-2. The 3D-printed PCL mesh acted to keep the artificial bone graft in a contained area, providing an organized scaffold for osteocytes. The PCL mesh was applied onto the bone defects simply and was easily fixed with simple sutures. Additionally, 3D printing enabled the mesh to fit defects with different sizes and lengths. Thus, there was no need to adjust the shape or volume of the applied polymer (Yu et al., 2020). Vancomycin-loaded in micro-arc oxidised (MAO) 3D printed porous Ti6Al4V scaffolds have been proved to have good osteogenesis and sustained vancomycin release properties, providing new way to treat complex bone infections. 3D-printed customized porous Ti6Al4V implants with interconnected macropores and high porosity can meet the requirements of osteoconduction and stability owing to their good flexibility, reproducibility, and cost-effectiveness. In a rabbit tibia osteomyelitis model, rabbits with vancomycin-loaded in MAO scaffolds showed the inhibition of bone infection and enhancement of osteogenesis, resulting in better outcomes than in the other groups (Zhang et al., 2020). In a study, a composite ink that combines HA and poly (trimethylenecarbonate) (PTMC) was used in a 3D printing process to produce scaffolds, which could be fabricated into patient specific dimensions and architectures with controlled porosities. The obtained composite ink was functionalized with a controlled release of BMP-2. *In vivo* experiments with the scaffold implanted in a critical size defect in tibia and cranium of a rabbit resulted in a conductive surface inducing bone formation and improved healing in both the tibia and cranium defect (Teotia et al., 2020).

Overall, these approaches will prove useful in expanding our understanding of how to design innovative bone substitutes for osteomyelitis treatment.

Innovative composites were constituted of bioactive glass, calcium-based composite, calcium phosphates, and polymers. Bone reconstruction of the dead space requires osteoconductive matrix, the structure of interconnecting porosity, the mechanical stability, and osteoinductive signaling. Osteoconductive matrix was designed based on the intrinsic properties (e.g., degradability, mechanical property and osteoconductive ability) of bioactive glass, calcium-based composite, calcium phosphates and polymers. Interconnecting porosity was essential for cell adhesion and fluid exchange. Mechanical stability was required for stabilization of the coagulum, temporal load transmission, and stress distribution. Nanofibers and nanoparticles were involved to enhance mechanical strength. Well-designed topology to mimic the haversian system of bone structure was explored to obtain mechanical strength. Osteoinductive signaling as BMPs was introduced into the composite for better bone reconstruction, as BMPs could regulate numerous processes of skeletal formation as well as induce the differentiation of bone cells.

## 9 Conclusion

**FIGURE 3:**
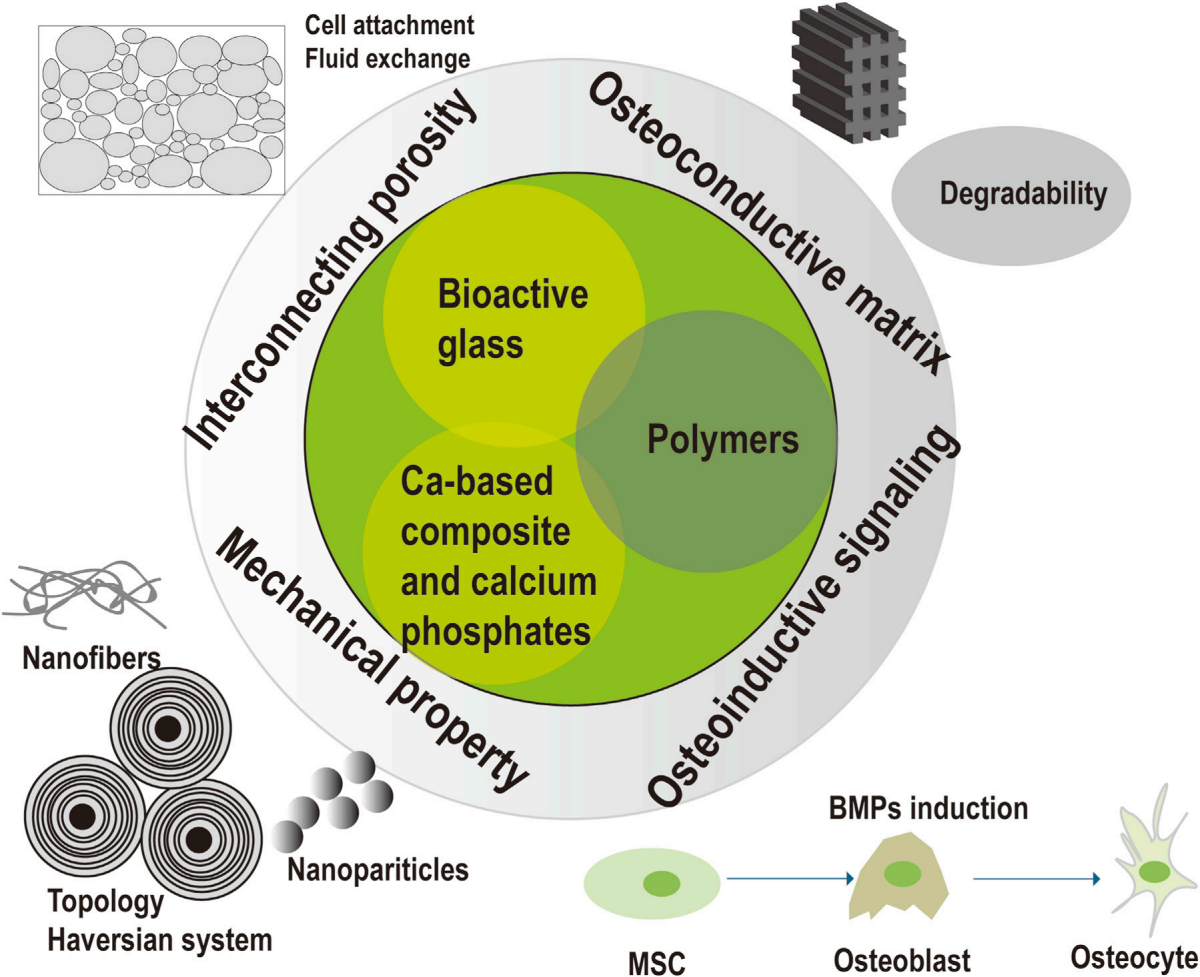
Bone reconstruction of innovative composites in the treatment of osteomyelitis.

The findings of this research provide insights into the likelihood of biodegradable substitutes incorporated with antimicrobial function for one-stage treatment of osteomyelitis. Current biomaterials can serve as both local carriers and osteoconductive scaffolds to eradicate the infection and simultaneously promote bone healing process, despite they may have some limitations. The postoperative complications for existing commercial bone substitutes contained reinfection, bone non-union, serum leakage and refracture, resulting in prolonged hospital stays, higher costs and more pain of patients. Hence, innovative biocomposites to solve the above problems includes composite design, structural modification, antimicrobial capability, processing technique, mechanical property, bioactive modification, and handling for optimizing infection eradication and bone reconstruction (see Figure 3). Optimized infection eradication is assumed to reduce the possibility of reinfection. Optimized bone reconstruction would avoid bone non-union and refracture. The balance between these factors remains controversial, thus extensive studies are required to definitively determine the benefits and limitations of these potential solutions.
